# Correction: Metabolic constraints drive self-organization of specialized cell groups

**DOI:** 10.7554/eLife.65803

**Published:** 2020-12-17

**Authors:** Sriram Varahan, Adhish Walvekar, Vaibhhav Sinha, Sandeep Krishna, Sunil Laxman

Varahan S, Walvekar A, Sinha V, Krishna S, Laxman S. 2019. Metabolic constraints drive self-organization of specialized cell groups. *eLife* **8**:e46735. doi: 10.7554/eLife.46735.

Published 26, June 2019

In the published article, Figure 2F inadvertently used a second image of the wild-type colony instead of the dark cell inoculated colony. A corrected figure 2 is now shown. The updated image reiterates that a wild-type *S. cerevisiae* colony on an agar plate that is inoculated using cells from an overnight liquid culture (leftmost colony in Fig. 2F) is similar in morphology to a colony inoculated using only dark cells taken from the former colony (bottom right colony in Fig 2F) and similar to one inoculated using only light cells taken from the former colony (top right colony in Fig. 2F). There is no change to the conclusions made in our paper.

The corrected Figure 2 is shown here:

**Figure 1:**
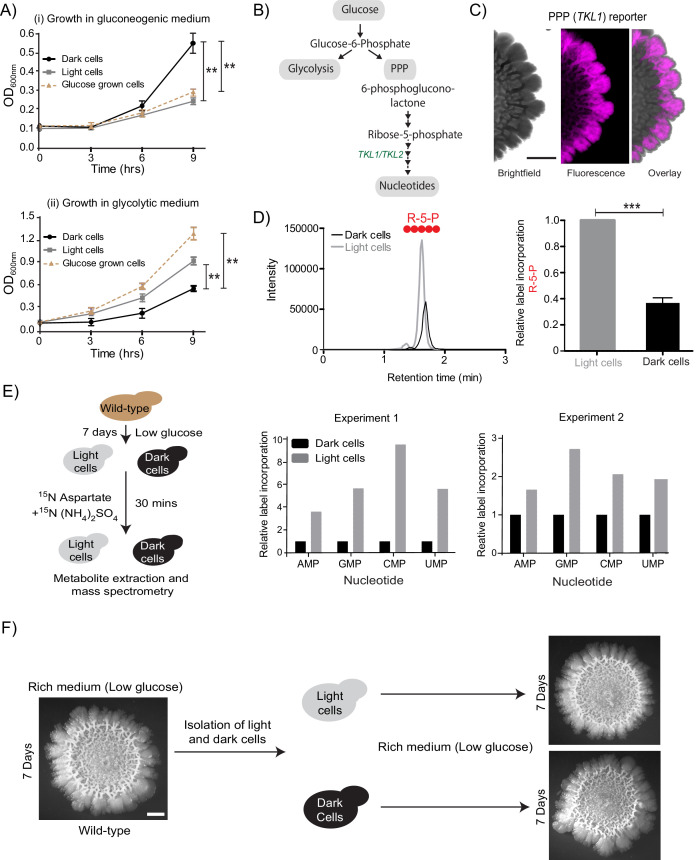


The originally published Figure 2 is also shown for reference:

**Figure 2:**
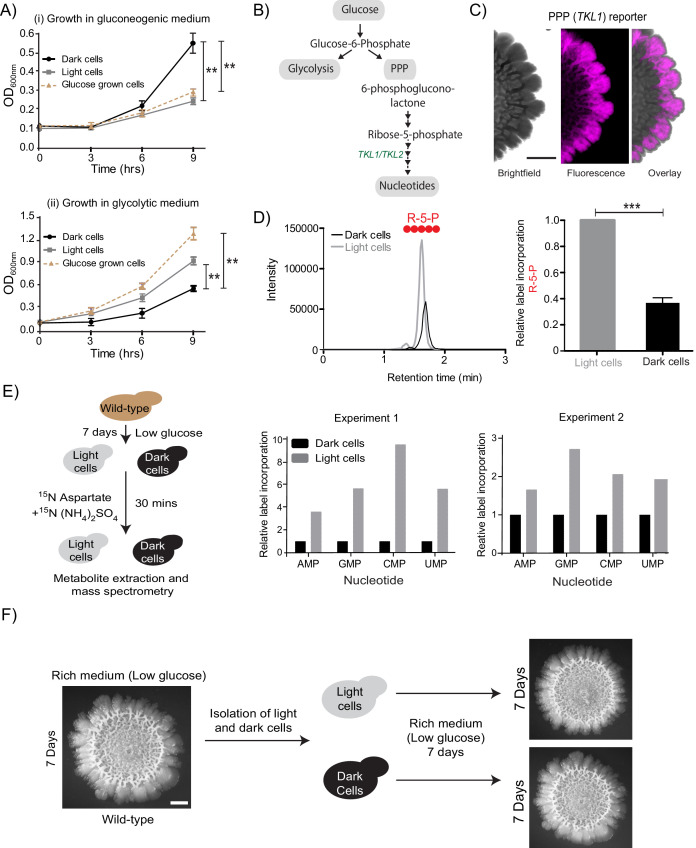


We have also updated figure legends of figures 3, 5 and 7 to indicate the intentional usage of a same colony image between figures, in order to provide a consistent representation.

Original sentence from Figure 3 legend:

(D) A simulation of the development of a wild-type colony, based on the default model developed. The inset shows an image of a real wild-type colony, which has developed for an equivalent time (~6 days).

Corrected sentence from Figure 3 legend:

(D) A simulation of the development of a wild-type colony, based on the default model developed. The inset shows an image of a real wild-type colony (same brightfield image used in figure 1B), which has developed for an equivalent time (~6 days).

Original sentences from Figure 5 legend:

(B) Comparative development of wild-type colonies with colonies…
(C) Visualization (left panel) and quantification (right bar graphs) of light cells in wild-type, *∆mal11*, …

Corrected sentences from Figure 5 legend:

(B) Comparative development of wild-type colonies (same image used in figure 1A) with colonies …
(C) Visualization (left panel) and quantification (right bar graphs) of light cells in wild-type (same image used in figure 2C), Δmal11, …

Original sentence from Figure 7 legend:

(A) Foraging response of wild-type cells and Δ*nth1* cells measured as a function of their ability to spread on a plate.

Corrected sentence from Figure 7 legend:

(A) Foraging response of wild-type cells (same image used in figure 1A) and Δnth1 cells measured as a function of their ability to spread on a plate.

